# Chloride Induced Reinforcement Corrosion in Mortars Containing Coal Bottom Ash and Coal Fly Ash

**DOI:** 10.3390/ma12121933

**Published:** 2019-06-15

**Authors:** Esperanza Menéndez, Cristina Argiz, Miguel Ángel Sanjuán

**Affiliations:** 1 Instituto de Ciencias de la Construcción Eduardo Torroja (CSIC), C/Serrano Galvache, 4, 28033 Madrid, Spain; emm@ietcc.csic.es; 2 Civil Engineering School, Technical University of Madrid, C/ProfesorAranguren, 3, Ciudad Universitaria, 28040 Madrid, Spain; cg.argiz@upm.es; 3 Spanish Institute of Cement and its Applications (IECA), C/José Abascal, 53, 28003 Madrid, Spain

**Keywords:** steel reinforced concrete, polarization, coal bottom ash, coal fly ash

## Abstract

Coal bottom ash is normally used as aggregate in mortars and concretes. When it is ground, its characteristics are modified. Therefore, the assessment of its long-term durability must be realized in depth. In this sense, an accelerated chloride ingress test has been performed on reinforced mortars made of Portland cement with different amounts of coal bottom ash (CBA) and/or coal fly ash (CFA). Corrosion potential and corrosion rate were continuously monitored. Cement replacement with bottom and fly ash had beneficial long-term effects regarding chloride penetration resistance. Concerning corrosion performance, by far the most dominant influencing parameter was the ash content. Chloride diffusion coefficient in natural test conditions decreased from 23 × 10^−12^ m^2^/s in cements without coal ashes to 4.5 × 10^−12^ m^2^/s in cements with 35% by weight of coal ashes. Moreover, the time to steel corrosion initiation went from 102 h to about 500 h, respectively. Therefore, this work presents experimental evidence that confirms the positive effect of both types of coal ashes (CBA and CFA) with regard to the concrete steel corrosion.

## 1. Introduction

Chloride penetration from seawater into concrete in coastal areas and the associated risk for reinforcement corrosion is recognized as the most important deterioration mechanism for offshore and coastal reinforced concrete structures worldwide [1,2]. Concrete pore solution provides protection to the reinforcing steel against corrosion by means of reinforcing steel passivation promoted by its high alkalinity. In chloride-containing environments, the passive layer is destroyed when the chloride ion concentration exceeds to a certain threshold value [3] in the vicinity of steel reinforcement and, therefore, the corrosion began. Then, the reinforced concrete service life can be divided into an initiation period and a propagation period [4]. The initiation one describes chloride ingress into the concrete and is ended by the reinforcement depassivation, which is followed by the propagation period beginning. The chloride amount associated with reinforcement depassivation has been extensively studied [5].

Several chloride threshold values have been proposed because it is affected by several interconnected parameters. Some of them depend on the type of cement used in the mortar and concrete, type of steel and steel/paste interface properties. Among them, the first one affects directly on the pore solution chemistry. Not only the type of cement, but also the concrete quality and concrete cover thickness influence the time needed to the critical chloride content to be reached at the steel reinforcement surface [6].

For electrical power generation by coal combustion to become sustainable, the reuse of coal combustion by-products such as bottom ash and fly ash is necessary. Blended cements made of coal fly ash present a beneficial effect since it has been acknowledged a more compact microstructure in mortars and concretes leading to lower chloride permeability [7]. However, a decrease of the pore solution pH was also found and, therefore, the chloride threshold value could be lower than in cement-based materials without pozzolanic materials. In any case, coal fly ash is frequently used in mortars and concretes exposed to chloride environments because the pozzolanic additions have a significant influence on the chloride ion transportation. This coal fly ash has a pozzolanic reaction and micro-filler effect, which is beneficial in improving the resistance of concrete against the ingress of harmful ions [7].

On the other hand, coal bottom ash (CBA) is regarded as a potential replacement for sand in concrete mixture. CBA can be used for construction purposes as a sand substitute [8,9] and for industrial purposes as alternative filter media [10] applications, amongst other uses. The amount of recycled aggregate is increasing in the world [11]. Given that, manufactured CBA sand with different sizes may be produced in a crushing plant. It is well known that size and particle size distribution will influence the material characteristics [12,13,14]. Other types of bottom ashes, from a municipal solid waste incinerator [15] or circulating fluidized bed combustion (CFBC) [16], are also reported in the literature. Among the various means of reusing coal bottom ash reported thus far, it is believed that CBA can be also utilized as a Portland cement constituent [17]. Thus, assessment of cement-based materials made of CBA mixes regarding corrosion performance must involve characterization of the penetration resistance against chlorides and the parameters governing the corrosion rate.

The influence of coal fly ash on chloride ingress and resulting reinforcement corrosion in concrete has been reported in many studies over the last decades [18,19,20]. Nevertheless, reinforcement corrosion studies have not been found with regard to coal bottom ash cement-based materials. The influence of coal fly ash in cement-based materials on the corrosion processes is mainly due to microstructural changes, lime consumption due to the pozzolanic reaction and binding capacity.

In this work, a standardized setup based on chloride ingress by applying an electrical field was used to study the initiation stage of chloride induced reinforcement corrosion in several mortar mixes made of coal bottom ash, coal fly ash and common Portland cement. Corrosion assessment of coal bottom ash in combination with coal fly ash in reinforced mortars was investigated.

## 2. Materials and Methods

### 2.1. Materials, Mix Proportions and Specimen Details

The cement used in this research work was a common Portland cement CEM I 42.5 N according to the European standard EN 197-1:2011 [21] produced by HOLCIM in Almería, Spain. Coal fly ash (CFA) Type F according to the American standard ASTM C618-15 [22] and coal bottom ash (CBA) were obtained from a Spanish power station (Carboneras, Spain). CFA and CBA were generated together in the same boiler of a coal-fired power plant, and then, chemical composition is expected to be quite similar. Chemical compositions of the cement, CBA and CFA, determined according to the European standard EN 196-2:2013 [23], are shown in Table 1.

**Figure 1:**
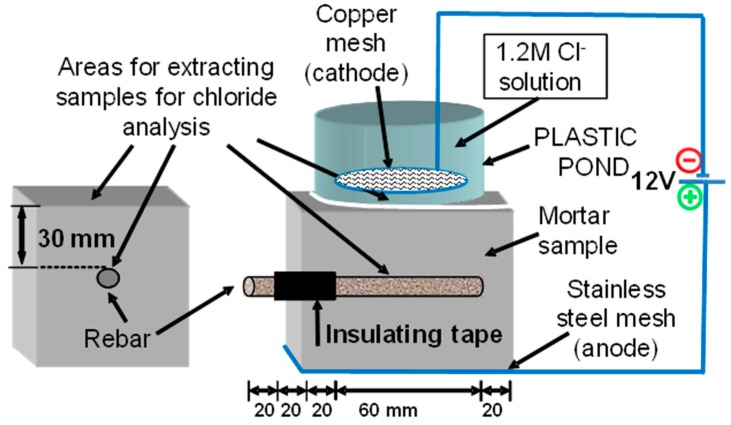
Geometry and embedded rebar and sensors (dimensions in mm).

Siliceous aggregates with a maximum size of 4 mm were used (provided by the IETcc-CSIC, Madrid, Spain). Distilled water was employed in the mixtures. The steel corrugated bars had a 6 mm nominal diameter and had been previously cleaned, to ensure a completely rust-free surface, in a 1:1 water-HCl solution containing 3 g/L of hexametilentetramine (corrosion inhibitor), rinsed in acetone, dried and then weighed. Their ends were covered with a plastic insulating tape leaving an exposed area in the end (Figure 1).

Coal bottom ash (CBA) and/or coal fly ash (CFA) was used as a partial replacement of cement at 0 wt %, 10 wt %, 25 wt % and 35 wt %. The detailed mix proportions of the mortar specimens are listed in Table 2. 70 mm × 70 mm × 70 mm size mortar cubes as shown in Figure 1 were cast with one steel bar inside. The direction of casting was horizontal to the rebars. The water-to-binder ratio for all tested mortar specimens was 0.50 and the binder-to-sand ratio was 1:3 (by weight). Table 2 shows the mortar dosage. The casting was done in two layers and the mass was consolidated by vibration. Then, the specimens were kept at 100% RH for 24 h and then, they were demolded. Later, the specimens were cured at 25 °C and at 100% of relative humidity for 28 days before placing the plastic pond on the top of the specimen (Figure 1).

### 2.2. Testing Procedure

The testing procedure was based on the method given in the Spanish standard UNE 83992-2 [24]. A pond with a 0.6 M NaCl and 0.4 M CuCl_2_ solution, which was prepared by dissolving 35.06 g of NaCl and 68.20 g of CuCl_2_∙2H_2_O in distilled water, was located on the top of the mortar specimen (Figure 1). The use of a copper chloride solution was to minimize the pH changes in the exposure solution [25]. A copper electrode (cathode) was submerged in the chloride solution. The anode (stainless steel mesh) was positioned on a water-saturated sponge at the bottom of the specimen. The stainless steel mesh (anode) and the copper electrode submerged in the chloride solution (cathode) were connected to the power source [24]. A 12 V difference in voltage was established between the two electrodes and the electrical current was measured immediately after the connection was made. Both parameters’ working voltage and current were recorded throughout the test. The initial and final values were used to calculate the initial resistance and the depassivation resistance according to Equations (1) and (2).

Re_initial_ = V/I_initial_ [Ω], (1)

Re_depassivation_ = V/I_depassivation_ [Ω], (2)

where R_e_ is the electrical resistance, in Ω; V is the voltage applied, in V; I_initial_ is the current circulating in the specimen 5–15 min after connection, in A; I_despassivation_ is the current circulating in the specimen shortly before the end of the test, in A.

Chloride ions penetrated the mortar cover from the top to the bottom face in an accelerated way induced by the electrical field. The corrosion process of the steel bar begins when the chloride ions reached the reinforcing bar. The test ended when the threshold amount of chlorides around the embedded reinforcement was achieved. Corrosion was considered to exist when the voltage, referred to the Ag/AgCl electrode, was less than or equal to −300 mV [24]. The trial was deemed to be over when two consecutive voltage readings more negative than −300 mV, with respect to the silver/silver chloride electrode, were recorded. Nevertheless, it has reported values less than −300 mV in other types of cement-based materials for steel corrosion onset [26].

The time needed for steel depassivation is related to the non-steady state diffusion coefficient, namely apparent diffusion coefficient, D_ap_. The depassivation time and the electrical charge, measured in coulombs, were recorded.

Electrochemical variables such as the corrosion potential (E_corr_) and the polarization resistance (R_p_) were monitored from the beginning until the end of testing in order to assess the long-term stability of the steel rebar. The corrosion current density (i_corr_) evolution was determined from R_p_ measurements as i_corr_ is inversely proportional to R_p_, according to Equation (3).

i_corr_ = B/R_p_ [μA/cm^2^], (3)

where i_corr_ is the corrosion current density, in μA/cm^2^; R_p_ is the polarization resistance, in kΩ∙cm^2^; B is the Tafel constant and usually it has a value of 26 mV.

A three-electrode arrangement was used to carry out the polarization resistance, R_p_, measurements: The steel rebar was the working electrode, the stainless steel mesh at the bottom of the specimen was used as the counter-electrode and a silver/silver chloride electrode was used as the reference electrode. The reference electrode was positioned in the solution when the current was shut off to take the measurements. Polarization resistance measurements were performed by applying a linear sweep with a sweep rate scan of 10 mV/min between −20 to +20 mV from the corrosion potential. Compensation of the ohmic drop was done at each measurement to remove the influence of the mortar resistance during the RP measurement [11]. The values were always measured when the voltage between the external electrodes was shut off. The “off-time” period ranged between 15 min and 4 h.

Finally, the specimens were split up to take mortar samples located around the reinforcing steel and at the surface in contact with the chloride solution to verify the extent of the corrosion. Then, the critical chloride concentration and the chloride concentration on the surface were determined by X-ray fluorescence, XRF, with a Bruker S8 TIGER (Bruker Corporation, Billerica, MA, USA), which is a WDXRF (wavelength dispersive X-ray fluorescence) spectrometer for elemental analysis. By varying the angle of incidence from 0° to 147.6°, a single X-ray wavelength was selected. Intensity and voltage were 80 mA and 100 kV, respectively [27]. A silver nitrate solution was also applied to one of the two parts of the concrete sample to determine whether the chlorides reached the steel.

### 2.3. Calculation of the Non-Steady State (Apparent) Diffusion Coefficient

Diffusion coefficient in natural test conditions was calculated by means of Equation (4).

(4) $$D_{\mathrm{ns}}\;=\;\frac{e^{2}}{2\;\times \;t_{\mathrm{lag}}\;\times \;\varphi }\;\left[{\mathrm{cm}}^{2}/s\right]$$

where D_ns_ is the diffusion coefficient in natural test conditions, in cm^2^/s; t_lag_ is the time to steel corrosion in accelerated test or time lag, in s; e is the cover thickness in the specimen to be tested, in cm; φ is the electrical field acceleration factor, which is calculated according to Equation (5) by using the normalized electrical field Δϕ, in V, following Equation (6).

(5) $$\varphi \;=\;\frac{z\;\times \;F}{R\;\times \;T}\;\times \Delta \phi =40\;\times \;\Delta \varphi \;\mathrm{for}\;22\;{}^{\circ}C$$

(6) $$\Delta \phi \;=\;\frac{\Delta V}{L}[V]$$

Here, L is the distance between electrodes (specimen thickness), in cm; ΔV is the voltage applied, in V; R is the ideal gas constant, in cal/(mol·K) (1.9872); F is the Faraday constant, in cal/V_eq_ (23,060); T is temperature, in Kelvin; z is the chloride ion valence (z = 1).

## 3. Results and Discussion

### 3.1. Depassivation Time and Non-Steady State Diffusion Coefficient

**Figure 2:**
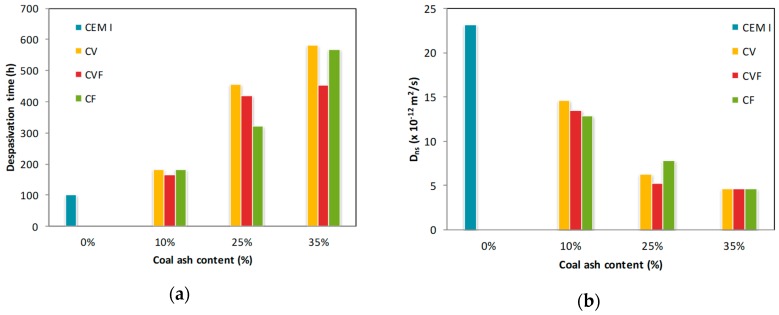
Time (**a**) and non-steady state (apparent) diffusion coefficient (**b**) vs. coal ash content.

Table 3 summarizes the parameters recorded during the test and Figure 2 presents the depassivation time monitored along the time and the calculated non-steady state (apparent) diffusion coefficient. The depassivation time increases with the percentage of coal ash in the mortar regardless the type of ash. With 25% and 35% of coal fly ash this effect is more pronounced than with the same content of bottom ash. Given that, coal fly ash apparently provides a better chloride penetration resistance.

The most common method widely used to assess the diffusion coefficient of chloride in cement-based materials is the measuring of the chloride profile after a time and fitting it in Fick’s second law of diffusion [14,28]. Such a coefficient could either overestimate or underestimate the time to initiation of corrosion due to the great influence of the surface chloride concentration on the result, which changes with time leading to errors in the prediction of the diffusion coefficient of chloride based on Fick’s second law.

Pore size redistribution by a pozzolanic reaction and higher chloride binding capacity of ash-cements reduces the non-steady state (apparent) diffusion coefficient in coal bottom ash and coal fly ash mortars by a factor of 0.44 and 0.37 times that of CEM I mortars in 10% ash replacement mortars; 0.66 and 0.73 in 25% ash replacement mortars; 0.80 and 0.80 times in 35% ash replacement mortars, respectively (Figure 2).

### 3.2. Critical and Surface Chloride Content

**Figure 3:**
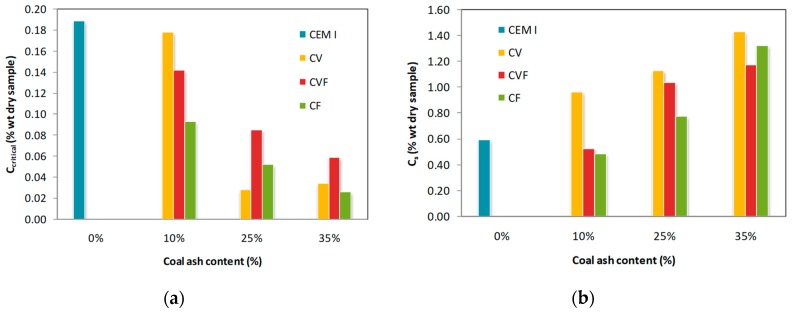
Critical (**a**) and surface (**b**) chloride content in the function of the coal ash content after testing.

The higher the coal ash content in the mortar, the lower the critical chloride content, C_critical_ (Figure 3). Chloride threshold level is affected by several factors [10,29], such as the chloride salt type [30], supplementary cementitious materials in the cement-based materials [11,31], origin of the chloride ions [32] and so on. Therefore, a wide range of threshold values has been reported. In particular, coal fly ash mortar has a lower chloride threshold level than that of the Portland cement mortar [33]. This fact may be attributed to the decrease of pH of the mortar pore solution due to the pozzolanic reaction of coal fly ash [34]. Consequently, the chloride amount needed for the passive film breakdown decreases. On the other hand, coal fly ash can improve the chemical binding ability of the mortars in some particular circumstances. Thomas [35] and Oh et al. [11] reported a decrease in the tolerable chloride content. Conversely, Alonso et al. [32] did not report any influence of coal fly ash content on the chloride threshold. On the other hand, longer depassivation times lead to higher surface concentrations as shown in Figure 3.

### 3.3. Initial and Final Resistance

**Figure 4:**
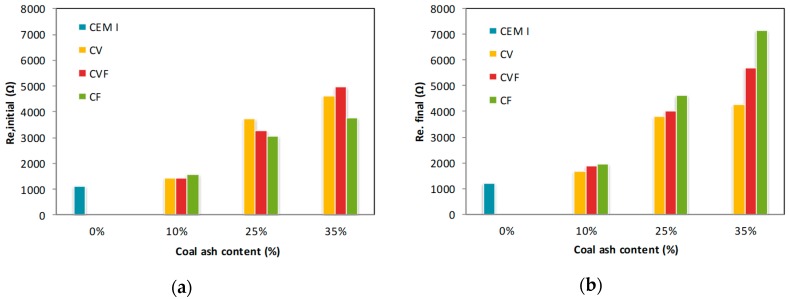
Electrical resistances measured in the bars embedded in blended mortars: (**a**) Initial, R_e,initial_ and (**b**) final, R_e,final_.

The high initial and final electrical resistances, R_e,initial_ and R_e,final_, measured in the bars embedded in blended mortars is attributed to their lower permeability and higher compactness (Figure 4) [12,13]. As expected, both initial and final resistance, R_e,initial_ and R_e,final_, increase with time because the hydration reaction produce a C-S-H gel that fills the pores (Figure 4). Moreover, the pozzolanic reaction produces more calcium silicates at longer times improving the coal ash mortars performance [16].

**Figure 5:**
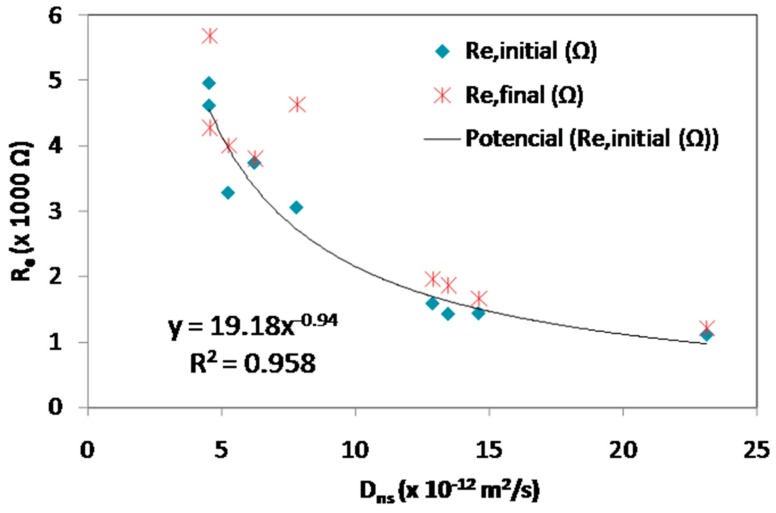
Initial and final resistance vs. non-steady state diffusion coefficient.

The initial resistance, R_e,initial_, results were fitted with the chloride non-steady state (apparent) diffusion coefficient ones (Figure 5). Then, the chloride apparent diffusion coefficient could be approximately estimated by means of Equation (7).

D_ns_ = (R_e,initial_ (Ω) − 4667)/190. (7)

### 3.4. Potential Monitoring

**Figure 6:**
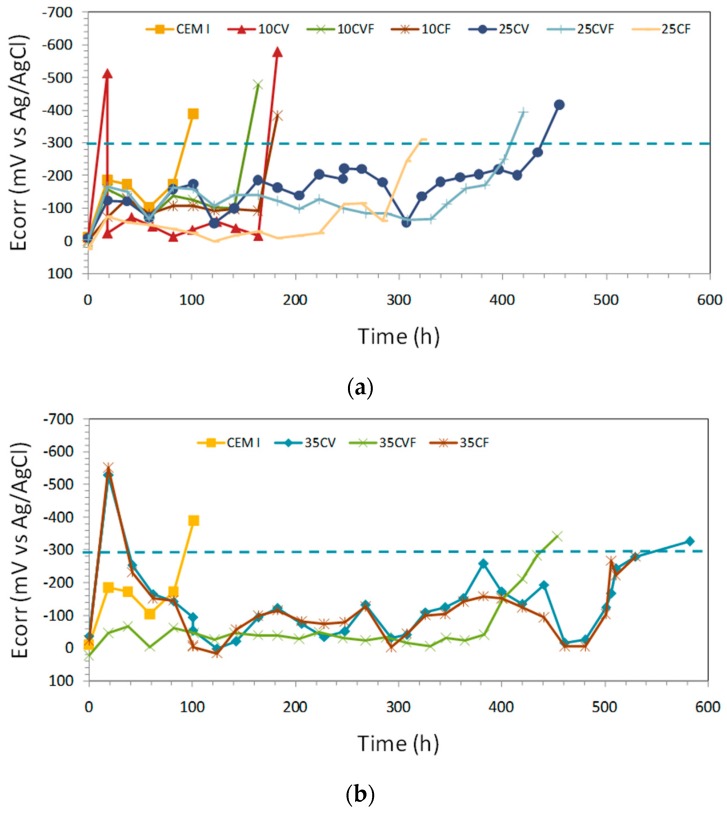
E_corr_ vs. time. The different lines per plot represent the samples per mix: (**a**) samples CEM I, 10CV, 10CVF, 10CF, 25CV, 25CVF, 25CF; (**b**) samples CEM I, 35CV, 35CVF, 35CF. The dashed line indicates the E_corr_ value for corrosion initiation (E_corr_ = −300 mV).

The steel potential evolution during the subsequent chloride solution exposure of the rebar is presented in Figure 6. In the passive state, the potential was generally stable with a tendency to decrease over time for all mixes. The readings were ranging from −200 to +23 mV Ag/AgCl during the first two weeks of testing corresponding to a state of passivity [24]. Then, large fluctuations between −60 and −230 mV Ag/AgCl were found until one month of testing.

Corrosion onset for the steel rebar embedded in the mortar specimen was apparent from a fall in potential. A reading more negative than −300 mV Ag/AgCl was considered as the corrosion onset threshold. Therefore, mortar specimens with fly ash showed a longer corrosion initiation period [6]. Given that, the more the coal ash amount, the longer the initiation period, regardless of the type of ash used, bottom ash or fly ash.

After 100 days, the potential of the reference mortar specimen without ashes, CEM I, became more negative than −300 mV Ag/AgCl, whereas the potential of the mortar specimens with 10% of ashes, 10CV, 10CVF and 10CF, reached more negative potentials than −300 mV Ag/AgCl after six months. Moreover, amounts of 35% of coal ash in mortars lead to longer initiation periods that ranged between 14 and 16 months.

Traditionally, corrosion potentials have been used as a complement to corrosion rate measurements in studies of steel reinforcement corrosion [36,37,38]. These measurements are merely qualitative, but are quite useful to detect electrochemical changes on a steel bar when monitored along the time. This technique is also valid to assess corroding zones by comparison with non-corroding ones in the same steel bar.

### 3.5. Corrosion Rate Monitoring

**Figure 7:**
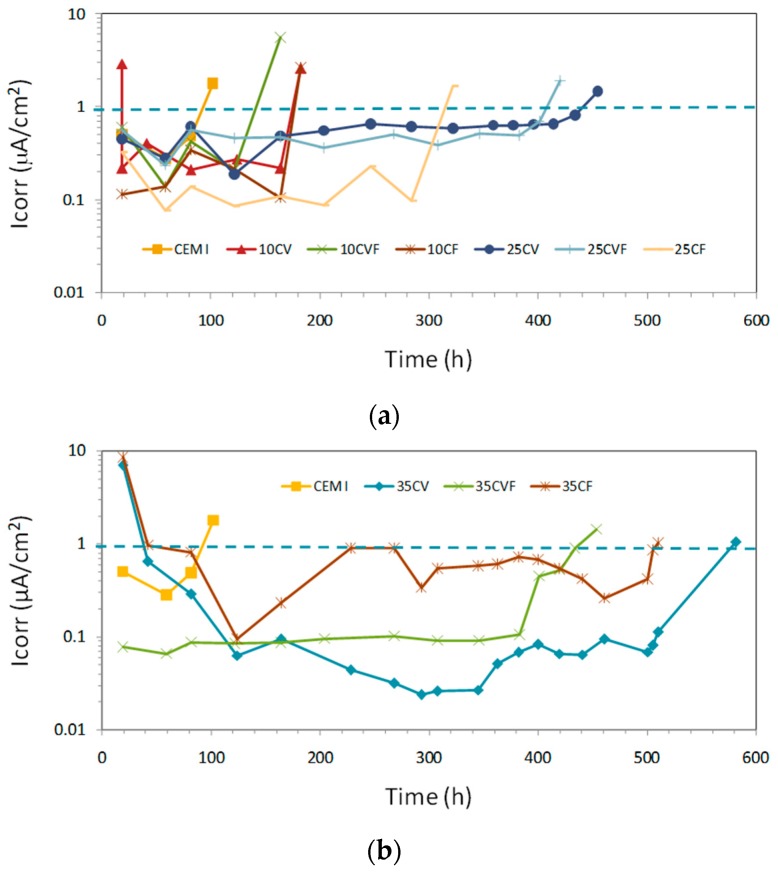
I_corr_ vs. time. The different lines per plot represent the samples per mix: (**a**) samples CEM I, 10CV, 10CVF, 10CF, 25CV, 25CVF, 25CF; (**b**) samples CEM I, 35CV, 35CVF, 35CF. The dashed line indicates the I_corr_ value for corrosion initiation (I_corr_ = 1 µA/cm^2^).

Steel potential monitoring could be not enough to assess the effect of the type and amount of coal ashes, since it is affected by a factor, which includes polarization by limited diffusion of oxygen among others [18]. Then, corrosion rate measurements were also undertaken. More stable corrosion rate readings than potential ones were registered throughout the test (Figure 7). According to reference [11], if the corrosion rate of steel in mortar becomes more positive than 0.1–0.3 µA/cm^2^, a significant corrosion process occurs. Within this research program, corrosion rates over 1 µA/cm^2^ were found at the end of the testing period.

As apparent from Figure 7, after 100 days of chloride exposure, stable corrosion initiated in only CEM I specimen. Later on, after 150 days corrosion initiated in three out of nine blended mortar specimens with 10% of coal ash independent of ash type. More than 300 days were needed in the rest of mortar specimens with 25% or 35% coal ash for corrosion onset. Thus, the samples with coal bottom ash and/or coal fly ash showed longer corrosion initiation periods than the samples without any ash (CEM I). This indicated that a substitution of coal bottom or fly ash increased corrosion resistance of steel in mortar. Moreover, the most important parameter influencing the corrosion onset is the amount of coal ash independent of the type of ash.

**Figure 8:**
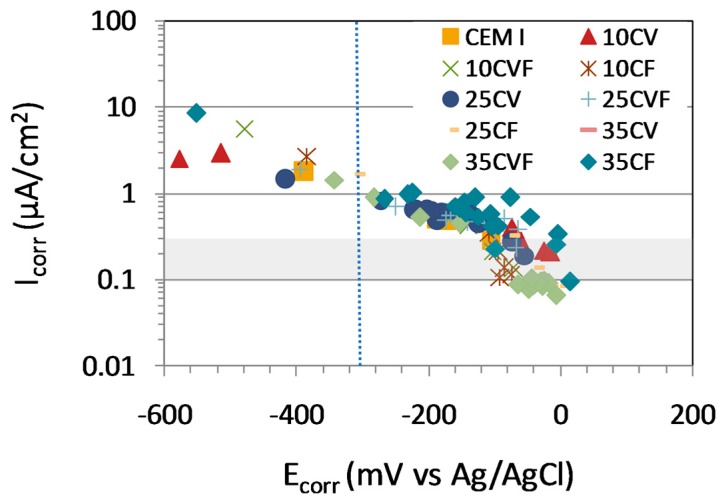
Relationship between I_corr_ and E_corr_ during the transition from a passive to an active state. Data of all the samples are considered before and after depassivation.

Corrosion rate evolution of the steel rebar embedded in mortar specimens was more stable than the corrosion potential. However, both of them showed the same trend. Therefore, Figure 8 shows a clear relationship between the corrosion rate and the corrosion potential, particularly at active corrosion states, regardless the content or type of coal ash. Higher fluctuations were recorded at low corrosion states. On the other hand, a clear correlation between corrosion rate and resistivity does not exist in chloride-induced corrosion [39]. The blue dotted line shows the limit of a high corrosion rate (1 µA/cm^2^).

Finally, it can be said that the results obtained from electrochemical tests showed that partial replacement of either coal bottom ash or coal fly ash has led to a reduction of corrosion rate and, therefore, an enhancement of corrosion resistance due to the decrease of chloride ions permeability.

### 3.6. Visual Examination

**Figure 9:**
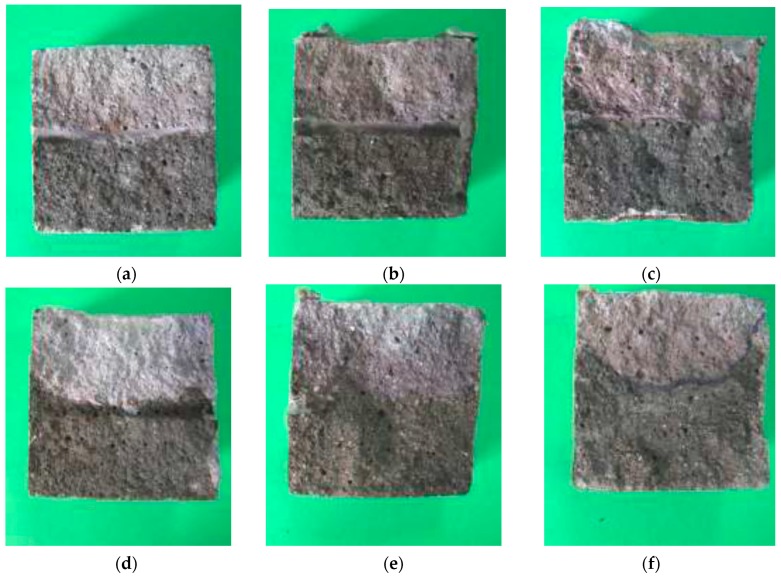

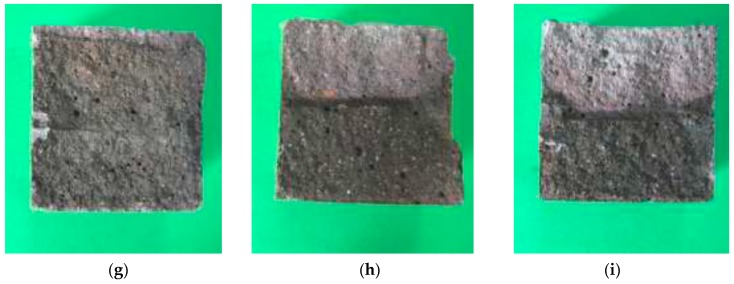
Sample of mortar extracted from the steel/specimen interface: (**a**) CEM I; (**b**) 25CV; (**c**) 35CV; (**d**) 10CVF; (**e**) 25CVF; (**f**) 35CVF; (**g**) 10CF; (**h**) 25CF and (**i**) 35CF.

After splitting the specimens and removing the steel rebar, corrosion was clearly visible in the cases where the electrochemical measurements had indicated depassivation (Figure 9). It was noticed the presence of rust spots on the steel. Red rust was found on both the steel and the mortar at the steel/mortar interface. However, red rust shown in Figure 9 could not show actual corrosion activities. Some works perform corrosion validation by means of measuring the mass loss of steel in corrosion [40] and chloride penetration in the mortar [41,42]. Nevertheless, the experimental procedure followed in this paper has been validated elsewhere [24].

## 4. Conclusions

The main conclusions are summarized as follows:

- Chloride diffusion coefficient in natural test conditions decreased from 23 × 10^−12^ m^2^/s in cements without coal ashes to 4.5 × 10^−12^ m^2^/s in cements with 35% by weight of coal ashes. Moreover, the time to steel corrosion initiation went from 102 h to about 500 h, respectively.
- Coal bottom ash and coal fly ash showed a similar corrosion performance in reinforced mortars. Both of them have a positive effect on the chloride resistance of the reinforced mortars. However, the higher coal ash proportion in the mortar, the lower critical chloride content, C_critical_, was found. This is explained by the lower hydroxyl concentration in blended mortars and, therefore, the lower Cl^−^/OH^−^ threshold value than in plain mortars.
- The most important parameter influencing the corrosion onset is the amount of coal ash independent of the type of ash.
- The results reveal that the experimental procedure used being accelerated appears a promising method to arrive at the chloride apparent diffusion coefficient, D_ap_, in mortars and concretes. It provided reliable information about the quality of the coal bottom ash investigated in this research program with regard to its durability.

## Figures and Tables

**Table 1:** Properties of used cements and ashes: Coal bottom ash and coal fly ash (%).

Compositions	SiO_2_	Al_2_O_3_	Fe_2_O_3_	CaO	MgO	SO_3_	TiO_2_	P_2_O_5_	Soluble Residue ^1^	Loss on Ignition
Cement	19.04	3.85	3.43	57.16	1.54	3.14	0.17	0.07	2.15	3.93
Bottom ash	48.12	25.55	5.86	7.07	1.28	0.15	1.5	0.96	81.24	1.85
Fly ash	46.84	26.66	4.72	5.55	1.33	0.37	1.5	1.03	76.00	3.63

^1^ Insoluble residue determined by the Na_2_CO_3_ method (European standard EN 196-2:2013).

**Table 2:** Mix proportions of coal bottom ash, coal fly ash and cement, CEM I 42.5 N.

Composition ^1^	CEM I	10CV	10CVF	10CF	25CV	25CVF	25CF	35CV	35CVF	35CF
Cement	100	90	90	90	75	75	75	65	65	65
Fly ash	0	10	8	0	25	20	0	35	28	0
Bottom ash	0	0	2	10	0	5	25	0	7	35
Sand	300	300	300	300	300	300	300	300	300	300
Water	50	50	50	50	50	50	50	50	50	50

^1^ CEM I is the cement without coal ashes; 10CV: 10% of coal fly ash; 10CVF: 10% of coal fly ash and bottom ash; 10CF: 10% of coal bottom ash;; 25CV: 25% of coal fly ash; 25CVF: 25% of coal fly ash and bottom ash; 25CF: 25% of coal bottom ash; 35CV: 35% of coal fly ash; 35CVF: 35% of coal fly ash and bottom ash; 35CF: 35% of coal bottom ash.

**Table 3:** Recorded for each mortar mix ^1^.

Code	t_lag_ (h)	D_ns_ (× 10^−12^ m^2^/s)	I_corr_ at t_lag_ (µA/cm^2^)	E_corr_ (mV)	C_critical_ (% wt Dry Sample)	C_s_ (wt % Dry Sample)	R_e,initial_ (Ω)	R_e,final_ (Ω)	Cover Thickness (cm)
CEM I	102	23.13	1.78	−389	0.19	0.59	1105	1212	3.10
10CV	183	14.61	1.18	−578	0.18	0.97	1432	1660	3.30
10CVF	164	13.47	5.51	−478	0.14	0.53	1423	1863	3.00
10CF	183	12.89	2.65	−384	0.09	0.49	1583	1960	3.10
25CV	455	6.24	1.46	−416	0.03	1.13	3738	3800	3.40
25CVF	420	5.26	1.91	−394	0.09	1.04	3279	4000	3.00
25CF	322	7.81	1.68	−310	0.05	0.77	3053	4633	3.20
35CV	582	4.55	6.94	−326	0.03	0.53	4615	4270	3.60
35CVF	454	4.55	1.43	−342	0.06	1.17	4959	5673	2.90
35CF	567	4.63	1.06	−331	0.03	1.32	3750	7143	3.27

^1^ t_lag_ is the time lag or the time to steel corrosion in the accelerated test; D_ns_ is the diffusion coefficient in natural test conditions; I_corr_ at t_lag_ is the corrosion rate measured at the time lag; E_corr_ is the corrosion potential; C_critical_ is the chloride critical concentration for the corrosion onset; Cs is the chloride concentration at the surface of the specimen; R_e,initial_ is the electrical resistance at the beginning of the test and R_e,final_ is the electrical resistance at the end of the test.
